# Effects of epigallocatechin gallate on the proliferation and apoptosis of the nasopharyngeal carcinoma cell line CNE2

**DOI:** 10.3892/etm.2014.2020

**Published:** 2014-10-15

**Authors:** WEIJUN ZHANG, PING YANG, FEI GAO, JIE YANG, KAITAI YAO

**Affiliations:** 1 Cancer Research Institute, Southern Medical University, Guangzhou, Guangdong 510515, P.R. China; 2 Department of Radiotherapy, Cancer Institute and Hospital, Guangzhou Medical University, Guangzhou, Guangdong 510095, P.R. China; 3 Department of Oncology, Dongguan Donghua Hospital, Dongguan, Guangdong 523220, P.R. China

**Keywords:** telomerase, telomerase reverse transcriptase, epigallocatechin gallate, nasopharyngeal carcinoma CNE2 cells

## Abstract

The present study explored the effects of epigallocatechin gallate (EGCG) on the cell cycle, proliferation and apoptosis of the nasopharyngeal carcinoma cell line CNE2 *in vitro*. The proliferation of CNE2 cells was detected using the cell counting kit-8 method. Cell cycle distribution and apoptosis were detected using flow cytometry. The human telomerase reverse transcriptase (hTERT) mRNA expression was determined using reverse transcription polymerase chain reactions. The protein expression of hTERT and Myc proto-oncogene protein (c-Myc) was observed using western blot analysis. EGCG inhibited the proliferation of CNE2 cells in a concentration-dependent manner (P<0.05) and blocked the cell cycle progression of the cells. In the low concentration (100 μg/ml) group, the cell cycle arrest showed a time-dependent manner. However, as the concentration increased and action time was prolonged, this time dependency became less marked. EGCG promoted the apoptosis of CNE2 cells in a time-dependent manner. In addition, EGCG downregulated the mRNA and protein expression of hTERT and downregulated the expression of c-Myc protein. Downregulation of the expression of hTERT and c-Myc was more evident in the high-dose group (200 μg/mL). In conclusion, EGCG has proliferation-inhibiting, cell cycle-blocking and apoptosis-promoting effects on CNE2 cells. EGCG may be developed into an auxiliary therapeutic agent for the treatment of nasopharyngeal carcinoma.

## Introduction

Nasopharyngeal carcinoma (NPC) is one of the most common types of malignancy that frequently occurs in South China and Southeast Asia. The development of NPC involves multiple genes and steps, among which anti-oncogene inactivation and oncogene activation play critical roles (1). At present, radiotherapy and chemotherapy are the primary treatment measures for NPC. However, these measures cause serious adverse reactions and have a tendency to induce multidrug resistance (MDR). Therefore, the need to find highly effective anti-NPC drugs that induce less-serious adverse effects is urgent. Considering the fact that the majority of antitumor drugs used in clinical practice are chemical preparations, which normally cause a number of toxic side-effects, research has instead started to focus on natural compounds. Studies have shown that natural components in certain plants exhibit antitumor effects, such as parthenolide in feverfew (chrysanthemum) and tea polyphenols (TPs) in green tea (2–3). TPs are the primary components in tea (particularly green tea), and catechin is an important polyphenol compound among TPs. Epigallocatechin gallate (EGCG) is an important variety of catechin. A study has shown that EGCG has a marked inhibitory effect on the cells of a number of types of human tumor, and that it induces cell apoptosis by regulating the activity of caspase through different channels (5).

As a broad-spectrum antitumor molecule, EGCG inhibits the proliferation of several tumor cell types (6–8). However, the biology underlying this effect, particularly on NPC, has been rarely studied. EGCG induces the apoptosis of the NPC cell line CNE2 through the mitochondrion-targeting signal transduction pathway (9). However, the underlying mechanisms of this process remain unclear, and such issues as the correlation between the epigenetic changes caused by gene methylation during the anti-NPC process of EGCG and its signaling pathways remain to be explored. To determine the anti-NPC mechanisms of EGCG *in vitro*, we investigated the effect of EGCG on human telomerase reverse transcriptase (hTERT), the telomerase in the NPC cell line CNE2, as well as on its gene expression. The aim of this study was to provide theoretical and scientific bases for new drug exploitation.

## Materials and methods

### Cell counting kit-8 (CCK-8) testing for the effect of EGCG on the proliferation of CNE2

NPC CNE2 cells were supplied by the Institute of Oncology of Southern Medical University (Guangzhou, China). The study was approved by the Affiliated Cancer Hospital of Guangzhou Medical University (Guangzhou, China) and conformed to the guidelines of the hospital’s Ethics Committee. The cells were grown in RPMI-1640 medium (HyClone Laboratories. Inc., South Logan, UT, USA) and supplemented with 10% fetal calf serum (FCS) in 5% CO_2_ and saturated humidity at 37°C. Cells with good morphology and growth were collected for experimental use. Cells treated with 50, 100, 150 and 200 μg/ml EGCG (95% purity; Chengdu Biopurify Phytochemicals Ltd., Chengdu, China) formed different experimental groups. Those treated with 0 μg/ml EGCG constituted the control group. The absorbance (A) values of all samples were detected at 12, 24, 48 and 72 h. Cell inhibition ratios were calculated based on the following formula:

$$\text{Cell inhibition ratio }(\%)=1-\frac{\text{A }(\text{experimental})-\text{A}(\mathrm{blank})}{\text{A}0\;(\text{experimental})-\text{A}(\mathrm{blank})}\times 100$$

Cells in the logarithmic growth phase were digested routinely with 0.25% trypsin (Gino Biomedical Technology Co., Ltd., Hangzhou, China) and then centrifuged to remove residual culture medium and trypsin. Subsequent to counting, the cells were homogeneously transferred to a 96-well plate. After 24 h, the cells were observed to exhibit good morphology and growth (they occupied 60–70% of the wells with ~3,000 cells in each) under an inverted microscope. EGCG at different concentrations was added.

CCK-8 tests were performed at different time-points. The media were replaced with 100 μl fresh media. Approximately 10 μl CCK-8 reagent (Gino Biomedical Technology Co., Ltd.) was added to each well and oscillated. The samples were stained away from light in a 37°C calorstat (Amersham Pharmacia Biotech Ltd., Little Chalfont, UK). The optical density (OD) value of each well was read at a wavelength of 560 nm (the enzyme-labeling instrument was a product of Bio-Rad, Hercules, CA, USA).

### Flow cytometry for the effect of EGCG on the cell cycle of CNE2 cells

Experiments were respectively conducted based on different time-points (12, 24, 48 and 72 h) at the same concentration (100 μg/ml) and different concentrations (50, 100, 150 and 200 μg/ml) at the same time-point (48 h). Cells in the logarithmic phase were digested with 0.25% trypsin and then centrifuged to remove residual medium and trypsin. Subsequent to counting, the cells were homogeneously transferred to 6-cm culture dishes. After 24 h, the cells were observed to exhibit good morphology and growth, occupying 60–70% of the dishes. EGCG at different concentrations was then added. The cells were collected and detected with a flow cytometer (Beckman Coulter, Miami, FL, USA) at different time-points. Between 10,000 and 20,000 cells were counted and their cycle was analyzed using the ModFit simulation software (Verity Software House, Topsham, ME, USA).

### Detection of rate of apoptosis

CNE2 cells were subcultured in RPMI-1640 medium containing 10% FCS in 5% CO_2_ and saturated humidity at 37°C. Cells with satisfactory morphology and growth were collected for experimental use and divided into experimental (200 μg/ml) and control (0 μg/ml) groups. EGCG at the corresponding concentration was added to the dishes. The cells were collected at different time-points (0, 12, 24, 48 and 72 h) for the detection of the rate of apoptosis.

### Reverse transcription polymerase chain reactions (RT-PCR) for the hTERT mRNA expression in CNE2 cells

Cells were cultured using the above-mentioned method and grouped based on different time-points (12, 24, 48 and 72 h, with the 0 h group as the control) at the same concentration and different concentrations (50, 100, 150 and 200 μg/ml, with the 0 μg/ml group as the control). EGCG at the corresponding concentration was added to the dishes. RNA was extracted at different time-points and integrity detection was performed.

### micro(mi)RNA RT

miRNA was reverse transcribed using an iScript Advanced cDNA synthesis kit (Bio-Rad), and the primers for miR-155 reverse transcription were designed using the stem-loop method. Total RNA was quantified with diethylpyrocarbonate (DEPC) liquid and each sample was diluted to 250 ng/μl. Two-step RT was performed with 20 μl reaction system.

At the first step, the miRNA-primer mixture (including 1 μg total RNA, 2 μl 0.5 μM RT primer and 15 μl DEPC H_2_O) was prepared and oscillated. The reaction system was incubated at 65°C for 5 min and then immediately cooled on ice for at least 2 min to form stem- and bulge-loops and template miRNA. Following specific binding, the miRNA-primer mix was formed. At the second step, 4 μl 5X iScript Reaction Mix and 1 μl reverse transcriptase were added to the miRNA-primer mix and oscillated. RT was performed at 25°C for 5 min and 42°C for 5 min, and then inactivation was initiated at 85°C for 5 min. The obtained products were five times diluted with ddH_2_O (20 μl RT product was diluted with 80 μl ddH_2_O) and then stored at −20°C.

Fluorescent quantitative PCR was performed using the SYBR method to detect the changes in hTERT mRNA expression. The reaction system contained 5.0 μl SYBR Premix DimerEraser (2X), 0.5 μl PCR reverse primer (20 μM), 0.5 μl PCR forward primer (20 μM) and 0.5 μl ROX Reference Dye II (Takara Biotechnology Co., Ltd., Dalian, China). Following oscillation, the system was transferred to an eight-tube PCR strip. The amplification conditions consisted of 95°C for 30 sec, 45 cycles of 95°C for 5 sec and 60°C for 30 sec and 72°C for 30 sec.

### Western blot analysis for the protein expression of hTERT and Myc proto-oncogene protein (c-Myc)

Cells in the logarithmic phase were digested routinely with 0.25% trypsin and then centrifuged to remove the residual culture medium and trypsin. Subsequent to counting, the cells were homogeneously cultured in 10-cm culture dishes. After 24 h, EGCG at 0, 50, 100, 150 and 200 μg/ml was respectively added to the corresponding dishes. The cells were further cultured in 5% CO_2_ and saturated humidity at 37°C for 48 h for total protein extraction. The extracted total protein was subpackaged into three tubes. Protein quantification was performed according to the instructions of a bicinchoninic acid (BCA) kit (Generay Biotechnology Co., Ltd., Shanghai, China).

Prepared BCA solution (~200 μl) was added to each well and then allowed to stand at 37°C for 30 min. The OD value of each well was read at a wavelength of 562 nm. A standard curve was drawn and the protein concentration was calculated.

hTERT protein expression following treatment with different concentrations of EGCG was analyzed using western blotting. SDS-PAGE gel was prepared. SDS-PAGE protein sample buffer (5X; Bio-Rad) was added to the protein sample according to a 1:4 ratio and then heated in a 100°C boiling water bath for 5 min for sufficient protein denaturation. Following cooling to room temperature, the protein sample was subjected to electrophoretic separation by SDS-PAGE for 90–120 min.

The damp-dry protein samples were transferred to polyvinylidene difluoride membranes and rinsed in Tris buffered saline with Tween 20 (TBST) cleaning solution for 1–2 min. Western blocking buffer was added for 60 min of blocking at room temperature. The blocked membranes were thrice rinsed with phosphate-buffered saline with Tween 20 (PBST) solution. PBST solution containing 1% skimmed milk was diluted with primary antibody (cysteine string protein antibodies; Shanghai Ruiqi Bio-Technology Co., Ltd., Shanghai, China) according to a 1:1,000 ratio. The membranes were then incubated with primary antibody at 4°C overnight. On the second day, the primary antibody was recycled and the membranes were thrice rinsed with TBST cleaning solution. PBST solution containing 1% skimmed milk was subsequently diluted with secondary antibody (goat anti-mouse immunoglobulin G whole serum; Beijing Bioss Biotechnology Co., Ltd., Beijing, China) according to a 1:2,000 ratio. The membranes were incubated with secondary antibody at room temperature or 4°C for 1 h. The secondary antibody was then removed and the membranes were thrice rinsed in TBST cleaning solution.

### Protein detection

The membranes were fixed with X-clips and developing reagent was prepared (the same volumes of solutions A and B). The reagent was dropped onto the membranes. X-ray photograms were mounted for 1 min exposure.

### Statistical analysis

All experiments were repeated three times. All data are presented as the mean ± standard error of the mean and analyzed using SPSS 15.0 software (SPSS, Inc., Chicago, IL, USA). One-way analysis of variance was performed for comparisons between groups. P<0.05 was considered to indicate a statistically significant difference.

## Results

### Inhibitory effect of EGCG on CNE2 cells

**Figure 1:**
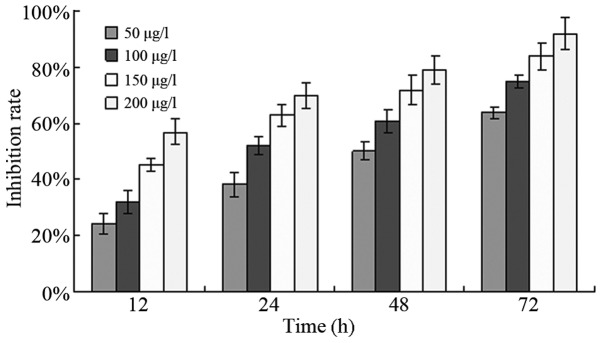
Effects of epigallocatechin gallate at different concentrations on the proliferation of CNE2 cells at different time-points. Time- and concentration-dependent effects were observed.

EGCG significantly inhibited the proliferation of CNE2 cells, showing time- and concentration-dependent manners (Fig. 1).

### Effects of EGCG on the cell cycle and apoptosis of CNE2 cells

**Figure 2:**
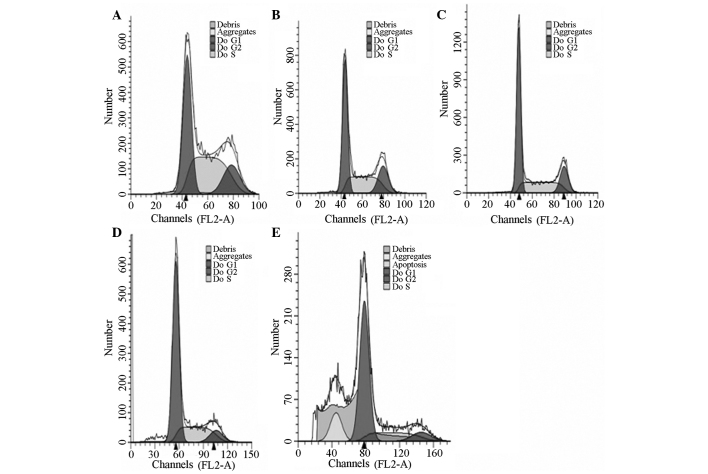
Effects of epigallocatechin gallate at the same concentration (100 μg/ml) on CNE2 cell cycle at different time-points, as determined using cell counting kit-8 tests. (A) Control group (0 h); (B–E) Experimental group at (B) 12 h, (C) 24 h, (D) 48 h and (E) 72 h.

**Figure 3:**
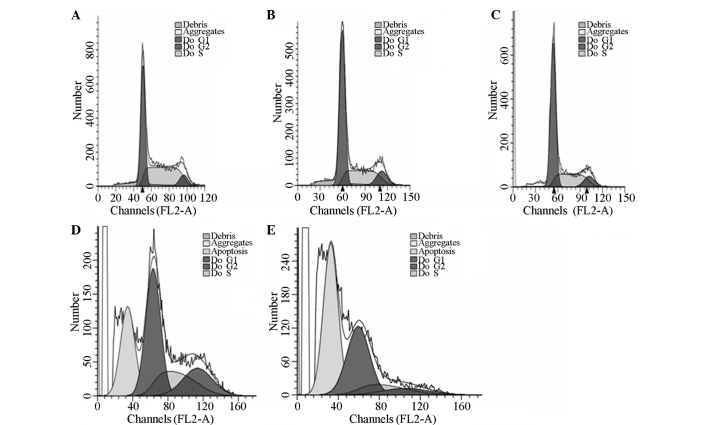
Effects of epigallocatechin gallate at different concentrations on CNE2 cell cycle at the same time-point (48 h), as determined using cell counting kit-8 tests. (A) Control group (0 μg/ml); (B–E) Experimental group at concentrations of (B) 50 μg/ml, (C) 100 μg/ml, (D) 150 μg/ml and (E) 200 μg/ml.

EGCG exerted a cell cycle-arresting effect on the CNE2 cells. In the low concentration (100 μg/ml) group, its blocking effect showed a time-dependent manner (Fig. 2). As the concentration increased and action time was prolonged, the effect became less evident, but the rate of apoptosis increased (Fig. 3).

**Figure 4:**
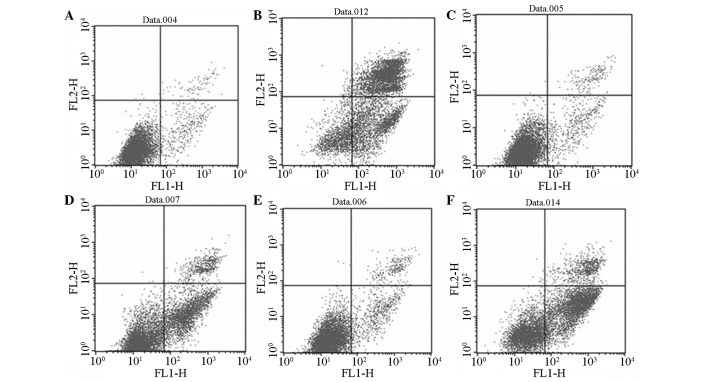
Effects of epigallocatechin gallate at different concentrations on CNE2 apoptosis at different time-points, as detected using flow cytometry. (A) 0 μg/ml and 24 h; (B) 200 μg/ml and 24 h; (C) 0 μg/ml and 48 h; (D) 200 μg/ml and 48 h; (E) 0 μg/ml and 72 h; (F) 200 μg/ml and 72 h.

**Figure 5:**
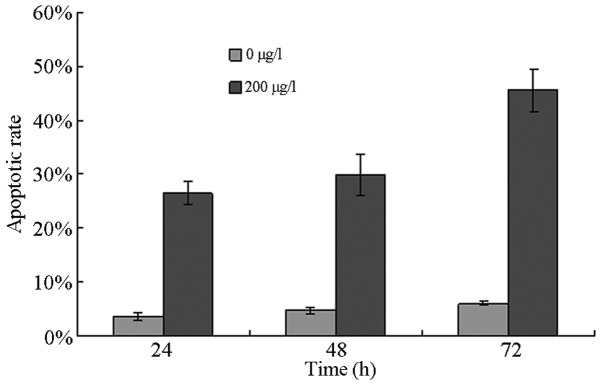
Comparisons among the effects of epigallocatechin gallate on CNE2 cell apoptosis. Data are presented as the mean ± standard error of the mean, n=3.

According to the results of the cell cycle detection, noticeable apoptosis appeared as the concentration increased (150 and 200 μg/ml) and the action time was prolonged. Based on this finding, the effect of EGCG on the apoptosis of CNE2 cells in the experimental (200 μg/ml) and control (0 μg/ml) groups was detected at different time-points (24, 48 and 72 h). The comparison of the apoptosis values (Fig. 4) between the experimental and control groups revealed differences at a significance of P<0.05. As the action time was prolonged, the apoptosis rate increased, showing a time-dependent manner (Fig. 5). This finding suggests that EGCG promotes the apoptosis of CNE2 cells in a time-dependent manner.

### Effects of EGCG at different concentrations and time-points on hTERT mRNA expression

**Figure 6:**
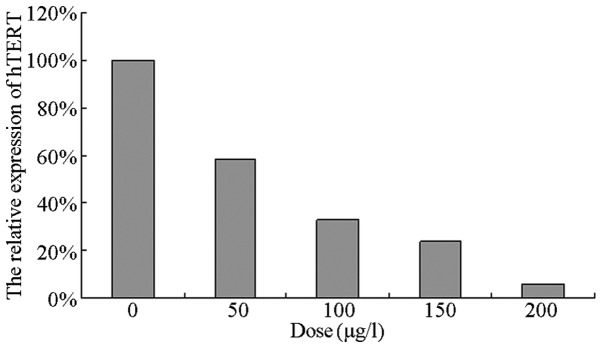
Epigallocatechin gallate downregulates hTERT mRNA expression at 48 h, showing a concentration-dependent manner. hTERT, human telomerase reverse transcriptase.

**Figure 7:**
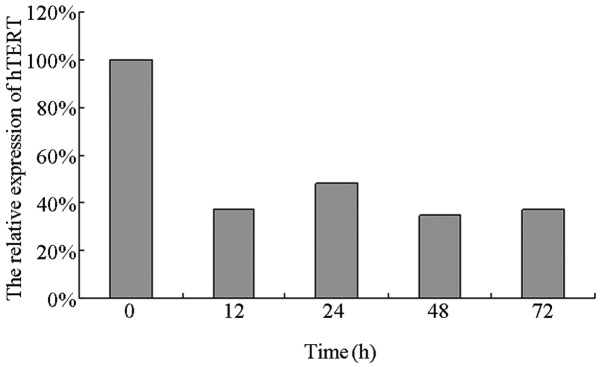
Effects of epigallocatechin gallate (100 μg/ml) on the hTERT mRNA expression in CNE2 cells (n=3). A time-dependent manner was observed. hTERT, human telomerase reverse transcriptase.

Agarose gel electrophoresis showed clear bands at 28S and 18S with all 28S/18S brightness ratios >2, which indicated that RNA was satisfactory in integrity without noticeable degradation. EGCG downregulated hTERT mRNA expression in CNE2 cells. At the same detection time-point (48 h), such an effect exhibited a concentration-dependent manner (Fig. 6), showing significant differences (all P<0.05). At the same concentration (100 μg/ml), the downregulatory effect showed a time-dependent manner (Fig. 7).

### Effects of different concentrations of EGCG on hTERT protein expression in CNE2 cells at 48 h

**Figure 8:**
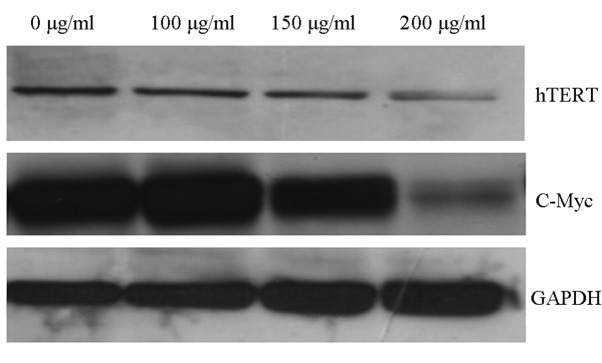
Epigallocatechin gallate inhibits hTERT protein expression in a dosage-dependent manner. hTERT, human telomerase reverse transcriptase; c-Myc, Myc proto-oncogene protein.

The effect of EGCG at different concentrations (0, 50, 100, 150 and 200 μg/ml) on hTERT protein expression in CNE2 cells was detected at 48 h. Cells treated without EGCG comprised the control group and GAPDH was taken as the reference protein. Relative protein expression levels were analyzed using western blotting. The results showed that EGCG inhibited hTERT protein expression, exhibiting a dosage-dependent manner (Fig. 8).

## Discussion

NPC has a five-year survival rate of 40–70%. This survival rate has been recently increased with the improvement and extensive application of the intensity-modulated radiotherapy and image-guided radiotherapy techniques. However, a number of patients still suffer from NPC recurrence or metastasis within five years. The primary reasons responsible for treatment failure are the radiation tolerance of tumors (10–13) and MDR (14–16). Therefore, the identification of highly effective, low-toxicity and radiosensitizing drugs is currently a research focus.

EGCG, the primary component in green tea, is a high-performance, nontoxic Chinese patent drug that has antitumor and immunoregulatory effects. The biochemical activity of EGCG has been proven; furthermore, a consensus on its tumor cell proliferation-inhibiting and apoptosis-promoting effects has been reached (17). In this *in vitro* study, the results showed that EGCG markedly inhibited the proliferation of CNE2 cells, arrested the cells in the G_0_/G_1_ phase and promoted their apoptosis. However, the pharmacology underlying these effects remains to be explored. Previous studies have suggested that the antitumor effect of EGCG may be associated with tumor cell proliferation-inhibiting enzymes (including urokinase plasminogen activator, insulin-like growth factor-1, matrix metalloproteinases, epidermal growth factor receptor, cell cycle regulatory protein and vascular endothelial growth factor), signal transduction pathway blockage (including the phosphatidylinositol-3 kinase, nuclear factor-κB, Ras/Raf/mitogen-activated protein kinase and activator protein-1 signaling pathways) and the induction of cell apoptosis pathways (18,19).

Telomerase activation promotes tumorigenesis by inducing cell proliferation and inhibiting apoptosis (20). hTERT is the catalytic subunit of telomerase, as well as the determinative factor for telomerase activation; therefore, hTERT expression is correlated with tumorigenesis and the malignant potential of tumors (21). Under the catalysis of hTERT, telomerase takes its own RNA as the template and synthesizes new sequences of 5′-TTAGGG-3′ to prolong the abbreviated telomeres during replication, thereby promoting cell proliferation and inhibiting cell apoptosis (22). Therefore, inhibiting the activity of hTERT at the gene or protein level can significantly inhibit the development of tumors. In this study, the mRNA and protein expression levels of hTERT were detected using RT-PCR and western blotting, respectively. The results showed that EGCG downregulated the activity of telomerase in CNE2 cells at the gene and protein levels, thereby inhibiting the proliferation of CEN2 cells and promoting their apoptosis. It is presumed that EGCG decreased hTERT protein expression subsequent to downregulating hTERT mRNA expression. However, considering that hTERT gene expression is regulated by numerous factors, multiple mechanisms underlying the apoptosis-inducing effect of EGCG on CNE2 cells may exist; as such further studies are required. In addition, the sensitization effect of EGCG on NPC remains to be explored.

EGCG may inhibit the transcription of hTERT mRNA and the translation of hTERT protein by downregulating c-Myc protein expression in CNE2 cells to decrease telomerase activity, thereby inhibiting the proliferation and promoting the apoptosis of CNE2 cells. Although the effect of EGCG on NPC and the mechanisms underlying the effect remain to be proved by animal experiments and *in vivo* studies, the results of the present study suggest that EGCG can serve as an alternative anti-NPC drug. Therefore, further studies are warranted.
